# Nursing students' motivation for, and experiences from, participating in a blended intensive programme on mass casualty incidents: a qualitative study

**DOI:** 10.1186/s12909-025-07425-y

**Published:** 2025-06-04

**Authors:** Andreas Kvam Sletnes, Cathrine Stranden, Alessandro Girotto, Elena Bandiera, Harald Geerts, Emanuele Galli, Jordy Kone, Malvin Torsvik

**Affiliations:** 1 Nord University, Høgskolevegen 27, Levanger, 7600 Norway; 2 San Raffaele University Hospital and Scientific Institute, Via Olgettina 60, Milan, 20132 Italy; 3 Windesheim University of Applied Sciences, Campus 2, Zwolle, 8017 Netherlands

**Keywords:** Mass casualty training, Exchange programs, Blended intensive programme, Short-term exchange programmes, Simulations, Simulation-Based learning, Team-Based learning, International collaboration, Education, Interdisciplinary, Students, Foreign

## Abstract

**Background:**

Student mobility programmes aim to enhance academic competence and personal growth. However, non-academic reasons are decisive for students’ decisions not to participate in exchange programmes abroad. Short exchange programmes are becoming increasingly popular among both institutions and students, addressing some of the non-academic reasons to not participate in exchange programmes. The aim of the study was to gain insight into nursing students’ motivation for, and experiences from, participating in a short-term exchange programme focused on mass casualty incidents.

**Method:**

A qualitative study was conducted using focus group interviews with 30 nursing students from Norway, the Netherlands, Spain, and Italy. The data were analysed using content analysis.

**Results:**

Three main categories were identified. These were “motivation”, “learning experiences” and “barriers and coping”. The two main motivational factors identified were the academic subject of mass casualty and the opportunity to experience international encounters. The learning experiences encompassed perspectives on nursing, cooperation and teamwork along with measurable academic outcomes related to the learning outcomes of the blended intensive programme. The participants expressed language and communication challenges and professional discrepancies as barriers in the learning process. Nevertheless, they also reported a feeling of coping and achievement after completing the programme and provided insights into various aspects of the learning climate during the blended intensive programme.

**Conclusions:**

Driven by students’ academic interest and motivation for international encountering, the BIP demonstrated educational and personal benefits, emphasizing the potential value of short-term international programmes in nursing education. The programme fostered important competencies such as emergency preparedness, intercultural communication and interprofessional collaboration. Coping with language barriers and professional discrepancies contributed to personal and professional growth.

**Supplementary Information:**

The online version contains supplementary material available at 10.1186/s12909-025-07425-y.

## Background

The globalization of healthcare is advancing, and nurses need knowledge and skills to care for an increasingly culturally diverse patient population [1–4]. It is no longer sufficient for healthcare professionals to focus solely on local or national challenges within healthcare [5]. International collaboration and perspectives are prerequisites for the development of global knowledge and for addressing global societal challenges [6]. Student mobility programmes enhance academic studies by introducing international influences, fostering cross-cultural understanding and broadening intellectual horizons. In nursing education, such activities play a pivotal role in developing international competencies and learning outcomes, which include the knowledge, skills and attitudes necessary for navigating and addressing the complexities of culturally diverse healthcare settings [7]. Integrating international activities into nursing degree programmes enhances students’ professional readiness. It also equips them to contribute effectively to global health initiatives, making these competencies increasingly critical in a rapidly globalizing world [8].

The Bologna Process is a European initiative involving 48 European countries, the European Commission and all major organizations for higher education in Europe. Through joint decisions, member countries work to reform their higher education systems to simplify international student mobility [8], highlighting the political importance placed on student mobility in Europe. The Norwegian government aims for half of all students graduating with a higher degree to have studied abroad [6].

Student mobility for three months or more has the highest political priority [8] and the highest priority in academic policies across educational institutions [9]. The longer the period of study abroad, the greater the personal development, formation, increased skills, cultural competence and language proficiency students will acquire [8, 9]. However, surveys indicates that non-academic reasons are decisive for students’ decisions not to participate in exchanges abroad. Key reasons given include the increased financial burden and the reluctance to be away from friends, partners or children for extended periods [10, 11].

Short exchange programmes are becoming increasingly popular among both institutions and students [12]. There are many advantages to shorter exchange programmes [13], addressing some of the barriers already mentioned [10, 11]. Erasmus + offers financial support for various types of exchange programmes, including Blended Intensive Programmes (BIP). These are short, intensive programmes that use innovative teaching and learning methods, combining digital and face-to-face collaboration among students from different countries. Students participate in a mandatory digital component that facilitates online collaboration before they engage in a short-term face-to-face mobility experience [14].

Four universities from Italy, Norway, Spain and the Netherlands have joined a consortium to run BIPs. The programme’s theme varies from institution to institution. At Nord University in Norway, the theme of the BIP is mass casualty incidents (MCI). In Norway, the national guidelines for nursing education state that nursing students should be familiar with measures to preserve life and health in major accidents and crisis or disaster situations [15]. MCIs are thus already part of the curriculum for nursing students in Norway, although not to the same extent as covered in this BIP. In alignment with the national guidelines, Nord University Campus Levanger has conducted annual full-scale MCI exercises since 2011.

It is of great importance that nursing students possess the knowledge, skills and general competencies necessary to address the challenges posed by mass casualty incidents, ensuring they are adequately prepared to respond when such situations arise. Learning nurse responses to mass casualty incidents can be achieved over a short period of educational exposure [16] and participation in MCI exercises has been shown to provide healthcare students with improved knowledge [17–19], practical skills [17, 19] and non-technical skills [20], as well as confidence in emergency preparedness [18, 21].

The BIP hosted by Nord University is a three-ECTS-credit programme with eight learning outcomes focused on developing essential skills, knowledge and competencies that enable students to perform effectively both independently and as part of a team in managing MCIs. In the digital part of the BIP, team-based learning (TBL) is used as a pedagogical method. TBL has proven beneficial in terms of clinical performance, engagement, self-study, learning ability and decision-making, especially for nursing students [22].

In the face-to-face part of the BIP, simulation-based learning (SBL) is widely used as a pedagogical method. SBL creates learning conditions designed to resemble real-world situations that may be encountered in the future [23]. SBL has become essential for modern nurse education [24]. Nevertheless, interprofessional simulations can be challenging to execute, as educational institutions often struggle to access participants from different health care fields for these kinds of simulation exercises. The number of studies demonstrating collaboration between institutions on interprofessional simulations are therefore limited [25], including those highlighting learning outcomes of such simulations. At the same time, even though they are becoming increasingly popular, there is also a significant lack of research on the topic regarding short exchange programmes [12]. To address this gap, this article explores the implementation of interprofessional SBL within short exchange programmes. By examining the nursing students’ motivation for, and experiences from, participating in a short-term exchange programme focused on MCIs, this article aims to shed light on the challenges and opportunities of interprofessional collaboration in such settings. The findings may contribute to the limited body of research on short exchange programmes in nursing education, particularly those incorporating SBL and interprofessional learning.

## Method

### Study design

A qualitative descriptive design was used, and focus group interviews were conducted to collect data [26]. Prior to this, a theme guide was developed (attachment 1).

### Participants

All students (*N* = 30) participating in the BIP were invited to participate in focus group interviews. Ten participants were from Norway, seven from The Netherlands, seven from Spain and six from Italy. The cohort consisted of second-, third- and fourth-year nursing students, all of whom agreed to participate in the study. Only three of the 30 participants were male.

### Intervention

The study was based on an intervention that consisted of a digital part and a face-to-face component. One month prior to the face-to-face component, five pre-recorded video lectures, along with lecture notes and other resource documents, were distributed to the students. Two real-time digital TBL sessions, each lasting two hours, were conducted three weeks and one week before the face-to-face component. The first TBL session primarily focused on the application of the ABCDE algorithm in the assessment and management of trauma patients, while the second session focused on mass casualty incidents, including mass casualty triage.

The face-to-face component lasted five days. The first day was dedicated to social and cultural activities. Day two involved skills training in essential techniques related to MCIs, such as mass casualty triage, trauma team operations, intravenous access establishment, cardiopulmonary resuscitation (CPR), tourniquet application, cervical collar placement and more. Day three included simulations with an emphasis on the primary survey using the ABCDE algorithm in a trauma team setting. On day four, students participated in a full-scale mass casualty exercise, where the BIP students acted as either casualties, facilitators or trauma team members. After participating in the exercise, debriefing sessions were held in groups based on institutional affiliation. The fifth and final day included a practical group examination and students’ evaluation of the entire program.

The MCI exercise on day four was conducted at an industrial shipyard with a crane collapse scenario resulting in 50 casualties. Police, ambulance services, the local hospital, the Norwegian Civil Defence, a rescue helicopter from the Norwegian Air Force, two municipalities, the inter-municipal emergency medical center, two fire departments and private partners participated. An estimated 400 individuals participated in the exercise, including around 150 nursing students, among them the 30 BIP students. Forty-three of the 50 simulated patients were nursing students, while the remaining seven were dummies used to simulate deceased individuals. The non-BIP nursing students that participated in the exercise received a three-hour long lecture on the topic and an hour long briefing about the exercise three days prior to the event. In the two days prior to the exercise, all participating nursing students who were not BIP-students had individual tasks to address to prepare them for participation.

All simulated patients had make-up applied corresponding to their injuries. A facilitator was assigned to each simulated patient. The facilitator informed the examining professional of non-simulable findings, such as pulse and blood pressure. The patients were triaged, treated and evacuated by the response personnel in accordance with the Norwegian national guideline for mass casualty triage. Casualties were transported from the scene by a rescue helicopter or ambulances. Each simulated patient had a predetermined destination: 13 were taken to the local hospital, while the remaining 30 were taken to a makeshift hospital established on the university campus, staffed by seven trauma teams, coordinators and triage nurses. Each trauma team on campus consisted of three nursing students and two fifth-year medical students. The medical students had one day of supervised training in trauma team management in the facilities of the local hospital prior to participating in the exercise. They took on the roles of anaesthetist or trauma leader, while the nursing students served as primary and secondary nurses, or as scribes. A faculty member was assigned to each trauma team, as well as to the coordinators and triage nurses. The faculty members facilitated learning by organizing short debriefing sessions within the trauma teams after each patient, ensuring improvement in the assessment and treatment of the next patient and the progression of the exercise.

### Data collection and analysis

Four focus group interviews were conducted, each lasting 35–55 min. Each focus group consisted of 6–8 BIP-students from all the participating BIP-institutions. A thematic guide focusing on the aim of the study was developed in advance to support the interviewers in the semi-structured interviews. Although a thematic interview guide was used, the students were encouraged to speak freely and to share what they found most meaningful following their participation in the exchange program. Each group was led by two faculty members (interviewer and moderator) from the BIP partner institutions, all but one of whom had participated in the programme. Due to the interview not being conducted in the students’ native language, language became a challenge in a few instances. This was addressed by the interviewer or moderator and other students offering clarification and support to ensure the students’ intended meaning was accurately understood. All interviews were conducted in person at the host institution’s facilities after the completion of the academic programme related to the BIP in the spring of 2024.

The interviews were audio-recorded, subsequently transcribed by AI and anonymized. The software used for the transcription was an AI-application incorporated in a secure data management tool called “Nettskjema” [27]. To verify the transcripts, the audio from the interview was listened to while the transcripts were being read and any transcription errors were corrected. A qualitative content analysis, without the use of any specific theoretical framework, following the methodological framework of Granheim and Lundman [28] was conducted by three authors (AKS, CS and MT) and then revised by HG and JK. Meaning-bearing units were identified, abstracted and condensed, leading to the development of subcategories and categories. The identification of meaning-bearing units and the development of subcategories and categories were initially carried out individually before the authors jointly discussed and analysed the data material. Any discrepancies during the coding process were handled with discussions among the coders until a mutual understanding was established.

### Ethical considerations

The study’s handling of personal data was submitted to, and approved by, the Norwegian Agency for Shared Services in Education and Research. All participants received both written and verbal information about the study. The written information included details about the study and data handling. It stated that participation was voluntary and that consent could be withdrawn at any time. Participants were assured that their contributions to the focus groups would not be identifiable, and informed consent was obtained verbally. The process of obtaining verbal informed consent was approved by the Norwegian Agency for Shared Services in Education and Research.

## Results

The aim of this study was to gain insight into nursing students’ motivation for, and experiences from, participating in a short-term exchange programme focused on MCIs. Aligned with this aim and in connection with the analysis, several subcategories were identified leading to three main categories (see Table 1). The main categories along with their corresponding subcategories are presented below.

**Table 1:** Table 1

Categories	Subcategories
Motivation	• Subject of interest • International encountering
Learning experiences	• Nursing perspective • Cooperation and teamwork • Academic outcome
Barriers and coping	• Communication barriers • Professional discrepancies • Coping • Learning climate

### Motivation

The students were interviewed about various reasons for wanting to participate in the BIP. The two main motivational factors identified were the academic subject of mass casualty and the opportunity to experience international encounters.

### Subject of interest

Most of the students stated an academic interest in the subjects of mass casualties and traumatology as their main reason for joining the BIP. They either had little to no prior experience with the subject in their nursing education and sought to gain it, or they were aware that their local nursing education programme offered limited opportunities to learn about it and wanted to supplement their regular studies. Some students had prior experience with the subjects during their education and expressed a desire to deepen their understanding.

Some students expressed a desire to test their ability to handle emergencies, while others specifically aimed to assess their capacity to manage acute situations to determine if emergency nursing could be a suitable career path upon completing their nursing education. Additionally, some students who were already considering a career in emergency nursing participated in the BIP to confirm whether this field aligns with their expectations and future career plans.

SBL was highlighted by a few students as a motivation for participating in the BIP. Several students noted that this type of learning activity was not available within their own country’s nursing education and viewed simulation as a valuable opportunity to enhance their practical skills in emergency nursing. Additionally, some students emphasized the chance to practice working in interprofessional teams, gaining a deeper understanding of how the emergency system functions as a whole, as a key motivation for joining the BIP: “*For me*,* the motivation was to learn more about trauma*,* to learn more about the procedures of a mass casualty*,* how different work-groups; ambulance*,* police*,* firefighters work at a place to see the whole perspective*” (FG3P6).

A few students mentioned that earning additional credits was a motivating factor for participating in the BIP, as well as the potential for enhancing their curriculum vitae (CV) and improve their qualifications for specific job opportunities.

### International encountering

Many students reported that the opportunity to go abroad and meet people from different countries was a key motivation for applying to the BIP. A few students emphasized that participating in the BIP made international travel more financially accessible. One student mentioned that having children made studying abroad difficult, but the one-week duration of the exchange programme made participation feasible.

Experiencing Norway and learning about Norwegian culture were also highlighted as motivational factors. Additionally, some students expressed interest in understanding healthcare systems, nursing practices, nursing education in different countries as well as the opportunity to share experiences and skills with peers from other countries.

Personal growth was frequently mentioned as a motivation for participating in the BIP. One student explained: *“I also think it’s good for everyone to go out there. I don’t know how to say it. But to do something like this*,* you experience a lot*,* you learn a lot*,* you meet new people. I think this is also fun*,* but also good for you. And to personally grow”* (FG2P6). Several students linked this personal growth to the experience of speaking a language other than their mother tongue. A few specifically highlighted communicating with individuals who have different native languages as a motivational factor and noted that gaining confidence in speaking English was a desired learning outcome.

### Learning experiences

Students highlighted the learning experiences acquired through their participation. These experiences encompassed perspectives on nursing, cooperation and teamwork, as well as measurable academic outcomes related to the learning outcomes of the BIP.

### Nursing perspective

Being part of an international group of students was perceived as a valuable learning experience in itself. One student highlighted the importance of understanding differences in perspectives and approaches to nursing, emphasizing that patients need to be treated as individuals: *“I think it’s an experience to learn from other people*,* from other countries. You realize in the end that each people has his own. […] People think different and you have to treat as individual ones” (FG4P3).*

Several students noted that playing the role of the patient during the main simulation exercise provided valuable insights into the patient’s perspective. These experiences were seen as beneficial for their future roles as nurses, enabling them to better understand how patients feel during emergencies. As one student expressed: *“It was really good to get to know how a patient feels. Because if I’m a nurse*,* if you never have been in an accident*,* you don’t know how you feel as a patient. So now I know what I should do as a nurse because I had the feeling of a patient. And that was pretty great” (FG3P1).*

Many students reported gaining a new perspective on the challenges of communicating with patients who do not understand the language used, emphasizing the importance of professionals speaking a language the patient comprehends. One student highlighted this experience, acting as a patient during the mass casualty simulation and preferred the professional to communicate in English: “*And that’s so strange because I think it was about me*,* but I didn’t know. So there is like a part that they can learn about*,* that they learn to talk English if there is a patient in English*” (*FG3P1).*

### Cooperation and teamwork

Most of the students highlighted the learning experience of cooperation and teamwork. Working in international groups emphasized existing differences in roles and responsibilities within medical teams across countries. Some students observed that the collaboration between nurses and doctors differed from what they were accustomed to in their own countries. For instance, one non-Norwegian student reflected: *“Like here in Norway*,* it’s like… the doctor-nurse relationship. We are used to that the doctor is always the leader. […] You collaborate more”* (FG2P8). These variations in roles and responsibilities were viewed positively, as they enhanced the programme’s learning outcomes. Students noted that discussing these differences and clarifying their roles within the team fostered learning through the exchange of knowledge and experiences. One student summarized this by stating: *“I believe that everyone of us will bring home something from the other colleagues from other countries*,* not just from the lessons*,* but also from the way we had to work and to cooperate with each other” (FG4P1).*

A few students emphasized the importance of remaining calm in stressful situations, recognizing it as a crucial factor for effective teamwork. Maintaining composure was also seen as essential for team communication, which several students identified as a key aspect of cooperation. The international nature of the groups, with students speaking different mother tongues, was described as a challenge for collaboration, as it required additional focus to ensure mutual understanding in the high-pressure context of emergency patient care. However, this challenge was also perceived as beneficial for the learning outcomes. One student expressed: “*I feel like maybe in the trauma room it’s even better to have a language barrier because you have to tell out what you want to do and then you maybe have to explain because not everyone understands and then you get to think more by yourself*” (FG3P5).

### Academic outcome

Academically, many students reported substantial growth in their knowledge of emergency nursing. Most of them indicated that they had gained significant insights into handling trauma and acute situations, utilizing the ABCDE assessment method, triaging patients and working effectively as part of a team. One student noted: *“Now I have more academic value around mass casualty*,* triage and especially trauma teams and how to work together”* (FG3P6). Some students also highlighted learning about the healthcare systems in different countries, as well as the nursing profession in those contexts.

Many students expressed appreciation for receiving feedback from both teachers and peers from different countries, recognizing that each individual brings a unique perspective, thereby enriching the learning experience. One student noted: *“You have some different kind of teachers also looking at you and maybe they see just slightly something else of you and you can learn about that” (FG2P6).* Some students emphasized that varying teaching methods, different approaches to solving tasks and the individual expertise of each instructor expanded their perspective on problem-solving. This led them to understand that there can be several appropriate approaches to nursing tasks, all ultimately achieving the same goal.

Some students also noted that the learning activities and physical surroundings contributed positively to the overall learning environment. One student highlighted the TBL as an effective introduction to the topic and a demonstration of the benefits of collaborative teamwork. Another student appreciated the online lessons, which provided essential content not covered at their home institution. One student also remarked the difference in resources and facilities at Nord University compared to their home institution, stating:” *I think coming here for me for my colleagues and also for my teachers was a really really really fantastic way to obtain those kind of plus that you have in your university*” (FG4P1).

### Barriers and coping

The participants identified language and communication challenges as well as professional discrepancies as barriers in the learning process. Nevertheless, they also expressed the feeling of coping and achievement after completing the programme and provided insights into various aspects of the learning climate during the BIP.

### Communication barriers

The students identified several aspects of language barriers as challenges during the BIP. For many, communicating in English was seen as a significant obstacle. While some students reported understanding English well, they struggled with speaking the language. Additionally, unfamiliar medical terms or medical terms known in the students’ native language but not in English were also identified as barriers.

Several students pointed out that being in a stressful emergency situation itself created a communication challenge, with the added pressure of having to speak English making the situation even more difficult to handle. A few students expressed discomfort during the simulated scenarios due to language barriers. Some Norwegian non-BIP participants in the MCI exercise, who did not speak English, further compounded these challenges.

Others, however, considered it appropriate for the first responders and the trauma team to communicate in Norwegian during the simulation, arguing that the healthcare team speaking the native language made the scenario more realistic: “*As it was in Holland*,* like you were coming to us*,* the professionals also were talking Dutch to each other. So you can’t understand them. So that makes it feel real*” (FG1P2).

### Professional discrepancies

Several students identified professional discrepancies as a barrier. These discrepancies were noted in various aspects of nursing practice, such as different units of measurement for blood samples (e.g., haemoglobin or blood sugar), the inclusion of a letter ‘X’ as a preliminary step before applying the ABCDE approach and variations in the positioning and movement of healthcare professionals’ bodies while performing CPR. These differences in patient treatment approaches created confusion and added to the stress already experienced due to language barriers. However, some students found these discrepancies interesting and believed that learning about them enhanced their academic experience. One student remarked: “*So they’re like different approaches and maybe you will try one of them and get more comfortable with the other method. It was like challenging but also useful*” (FG4P1).

### Coping

Many students reported an increase in self-confidence, both academically and personally, after completing the BIP. Most indicated enhanced language skills and a greater confidence in speaking English. Several students noted that overcoming the challenge of the language barrier contributed significantly to their confidence by the end of the programme. One student shared: “*I was afraid to talk English and now I’m just talking English and I’m making jokes in English and it’s all okay. So that’s funny how you build up your confidence and this week will really help with it*” (FG3P1).

Some students mentioned feeling insecure about handling emergency situations before participating in the BIP, but they became more confident in managing such situations as the programme progressed. One student emphasized that confidence in one’s abilities is essential to being a nurse. Another student reflected: *“I never knew anything about mass casualty incidents*,* ABCDE and so on. But when we started the simulations here and the lessons and the trainings*,* I got more comfortable and secure day by day” (FG4P1).*

Several students also described personal growth as a result of participating in the BIP. Learning to work collaboratively in a team and managing challenging patient scenarios in a foreign country, where they did not speak the local language, contributed to increased coping skills. As one student expressed: *“I wanted to learn how to manage this type of situation*,* how to treat the patient and how to collaborate with the team. And I think that we did it. I did it. And seeing how it was this experience*,* how I grew*,* how much I learned*,* I feel lucky” (FG2P4).*

### Learning climate

The students reported aspects of the learning climate that also can be linked to their coping. Making mistakes in a supportive learning environment was seen as an important aspect of personal growth, as one student noted: “*You make a lot of mistakes*,* but you learn from it. And that’s when you grow as a person*” (FG2P5).

Several students mentioned that the teachers’ approach to managing and organizing learning activities contributed to creating a safe learning environment. They highlighted the teachers’ calm demeanour, openness to questions and the absence of judgment when students lacked knowledge. One student shared: “*I felt really safe to ask questions if I didn’t know anything or something*,* and that you don’t get judged if you didn’t know something*,* because everyone is really eager to learn*” (FG1P6).

Additionally, many students stated that getting to know their peers outside the classroom, experiencing local culture and socializing in the evenings, positively influenced the learning climate on campus. Some students initially felt anxious about their knowledge or practical skills, worrying they might not measure up to their peers. However, personal interactions with other students made teamwork easier, and SBL became a more enjoyable and productive experience. One student remarked: “*We had the chance to know each other outside the university*,* like having fun together and so on and it was fine because we get to know really*,* not so close but however*,* we got to know each other and then while working on scenes or simulations we had that kind of link that allowed us to work along together quite well*” (FG4P1).

## Discussion

### Motivation

Students were motivated to participate in the BIP because of their interest or previous experience in emergency care, or because of limited exposure to it in their education, with the aim of acquiring knowledge aligned with their professional goals in emergency care. This motivation is further supported by the fact that mass casualty management has been under-represented in the undergraduate nursing curriculum, despite its growing recognition as a critical component in preparing future nurses to respond effectively to large-scale emergencies and enhance disaster preparedness [16]. Some students had prior experience with the subjects during their education and expressed a desire to deepen their understanding, which aligns with previous findings that students who seek interprofessional learning experiences may already hold a positive attitude towards interprofessional collaboration [29].

Students viewed the BIP as an investment in their future careers, recognizing that participation enhances their competitiveness in the job market. Study abroad programmes are increasingly valued in a globalized job market, as they can offer a distinct advantage. The potential for future employment opportunities has been highlighted in previous studies among undergraduate and graduate students [30, 31]. However, few studies have evaluated the tangible impact of study abroad programmes on career progression [32].

Many students were motivated by the opportunity to meet international peers and experience Norway, which is more than just a by-product of the BIP. Intercultural encounters are seen as an important mean of fostering cultural awareness and contributing to personal growth and professional identity development [33]. The intercultural encounter seems to develop collaborative international partnerships with mutual benefits for all participants, including the host students [34].

Financial barriers, family responsibilities and job obligations have been identified as the most common barriers to participating in study abroad programmes [30]. Offering short-term student exchange opportunities may appeal to a broader range of students by providing a more flexible timeframe and lower costs [35]. This underscores the importance of thoughtful programme design, which accommodates the diverse needs and circumstances of students, thereby facilitating greater participation. Previous research has also noted language barriers as a significant deterrent to applying for study abroad programmes [36]. It is therefore noteworthy that, in this study, participants identified the challenge of engaging with a different culture and language as both a barrier and a motivating factor.

### Learning experiences

The students undertook various roles within the main MCI exercise. Playing the role of a patient was, despite its apparent lack of popularity among the students, ultimately found to yield significant learning outcomes. Several studies support that playing the simulated patient role is building important insights into the patient’s experience of disease and care given [37]. “Walking in the shoes of the patients” [38] led to a nuanced understanding of how patients may experience a casualty situation, which broadened the students’ nursing perspectives. Empathy may be evoked by a change in perspective when a nursing student experiences the nurse-patient relationship from the bed [39]. Empathy is a fundamental competency for nurses to possess in order to meet the needs of their patients and a crucial component of professional nursing care [40].

In the role of a patient, the students also experienced how lack of understanding the health professionals’ communication intensified the frightening situation of being injured in an MCI. This experience highlighted the nurse’s responsibility to ensure clear communication, preferably in English, when the patient does not speak the native language. Participating in the BIP provided students with insights into the significance of communication as a fundamental aspect of professional care and patient safety, similar to other studies [41]. In an increasingly globalised world, where nurses work and people travel across borders, there is a growing need for bilingual nursing competency [42].

Students noted cross-country differences in medical team roles, especially nurse–doctor collaboration. These were seen as valuable learning outcomes of the BIP. This observation aligns with previous research describing that simulations provide an ideal opportunity for practicing interprofessional cooperation, thereby increasing participants’ confidence, knowledge and appreciation of the differences and similarities between various professions [16, 43]. Interprofessional competencies provide patient safety [44], and interprofessional education between nurses and medical students is a crucial prerequisite for improving patient-centered care [45]. Furthermore, engaging in interprofessional simulation has been shown to lead to an enhanced respect for each other’s disciplines and create a positive impression of the other profession [25].

Most students also expressed that collaborative learning improved their efficacy as team players as well as their communication with colleagues and patients, which aligns with previous findings [46]. Students viewed cooperation and communication as essential for teamwork and patient care, aligning with Johnson and Johnson’s [47] emphasis on communication for team productivity. Additionally, students highlighted the importance of remaining calm in stressful situations to achieve effective teamwork and successful communication. Acting under stress, especially when communicating in a non-native language, required additional focus to ensure mutual understanding and correct patient treatment. This extra challenge was described by participants as a positive learning outcome. An important aspect of handling emergency situations as a nurse is the ability to provide patient care in a chaotic environment. SBL is effective for creating such chaos [16]. By incorporating simulations of varying complexity throughout the BIP, students were able to practice nursing in realistic environments, which helped them manage the chaos of a realistic MCI exercise at the end of the course. Students in this study reported increased confidence in speaking English overall, which likely facilitated professional cooperation and teamwork in English.

WHO [48] suggests that several educational approaches including digital techniques could be used to improve the learners’ skills and competencies in practical learning about mass casualty management. In-person learning, small group learning, case-based discussions and role-playing or simulations are highlighted as appropriate learning activities. There is limited evidence to the effectiveness of mass casualty education courses, and many of the courses address a multidisciplinary approach for already trained professionals and are therefore not tailored to address the learning needs of nursing students [16]. Most of the students who participated in the BIP expressed a significant academic outcome. This suggests that the BIP may be a successful educational model for developing students’ theoretical knowledge and practical skills in mass casualty management.

There is a growing body of evidence supporting the effectiveness of SBL, particularly in medicine and nursing education [49–52]. Simulation allows participants to develop a broad range of practical clinical skills, including communication in clinical contexts, thereby bridging the gap between “knowing” and “doing” [49]. The BIP students emphasized several pedagogical aspects contributing to their academic outcomes, including receiving feedback from both national and international teachers and peers, learning and practicing practical skills and acquiring theoretical knowledge on the topics included in the BIP. This aligns with research highlighting the importance of educational feedback, repetitive practice and curriculum integration as key features to ensure the educational effectiveness of SBL [50].

The observed positive academic outcomes can also be linked to the students’ level of psychological preparedness. As noted by Humphreys, “preparedness” is one of three essential steps for successful learning through simulation [16]. In the BIP, psychological preparedness was fostered through a combination of digital theoretical sessions on the subject matter and activities designed to enhance collegial trust among students. This included group cooperation and team formation during practical learning sessions and small-scale simulations preceding the main exercise. Furthermore, the programme’s social and cultural content seem to have contributed to a safe learning climate. Starting the face-to-face BIP week with a full day of non-academic activities allowed students and teachers to build rapport, which students reported as contributing to their psychological preparedness.

SBL in nursing education is effective due to four key factors: the development of technical proficiency through physical practice and skill development; hands-on assistance from expert teachers providing tailored feedback; situated learning within the context of simulated scenarios; and the incorporation of the affective (emotional) aspects of learning, which are activated through interaction [49]. Thus, the students’ reported positive learning outcomes can be attributed to the combined learning activities integrated throughout the BIP, with a particular emphasis on the simulations. Motivation is described as a key factor that influence academic performance [53]. Students’ academic outcome can therefore also be connected to their motivational aspects of joining the BIP.

### Barriers and coping

The language barriers were perceived by the students as the greatest challenge associated with participation in the BIP. General language barriers in international exchange programmes for nursing students are a well-known challenge [54–57]. Both struggles with the English medical terminology and abbreviations [56] and the fact that stress exacerbates language challenges has also been previously described [58]. Language barriers have the potential to impact students’ experiences and learning outcomes [58], and can negatively impact students’ ability to collaborate [54, 59]. Nevertheless, language barriers are not necessarily exclusively negative. Language barriers can foster cognitive flexibility and enhance students’ communication skills as they navigate and adapt to new linguistic environments. Furthermore, these barriers can enrich students’ experiences and better prepare them to work in globalized professional settings [60]. Students who encounter language barriers often develop strong problem-solving skills as they find innovative ways to communicate [61]. Although language challenges can negatively impact students’ ability to collaborate [54], overcoming these challenges can lead to stronger academic and social bonds among the students [62] which was also demonstrated in this study.

In addition to language barriers [54, 63–66], the literature describes cultural differences [54, 63, 65, 67], different nursing practices [63–65], differing role expectations [63, 68] and inadequate support [67, 69] as barriers in exchange programmes. Previous studies also describe different nursing approaches to patient care as challenging within exchange programmes for nursing students [54, 70], suggesting professional discrepancies as a barrier. This aligns with the students in this study, who describe discrepancies in the practical execution of procedures. In addition to constituting a barrier, some students expressed observing and discussing these different approaches as useful. Observing various approaches can better equip students for future nursing practice by providing them with a broader experiential foundation. However, some students might struggle to benefit from observing different approaches, as they may lack the knowledge and experience needed to evaluate and compare the methods. To support the learning process in this BIP, the presence of academic staff was therefore of importance.

The students reported the teachers’ calm manner and approach to managing learning activities, as well as their openness to student questions, as a major contributing factor to a safe learning climate. A learning environment free from intimidation, sarcasm and punitive measures is crucial for allowing students to focus on the learning task rather than succumbing to anxiety or a desire to disengage [71, 72], and fosters positive attitudes and motivation towards learning [73]. The emotional context of learning situations is closely linked to Bandura’s concept of self-efficacy, which refers to students’ belief in their ability to succeed and solve tasks [74]. A student’s self-efficacy influences whether they approach a task with confidence or anxiety [73]. Previous research has shown a significant increase in self-reported perception of self-efficacy development in nursing students following participation in a trauma simulation [59] and a safe learning environment is widely recognized as a fundamental cornerstone for successful SBL [75, 76].

A supporting learning climate, facilitated by positive teacher-student interactions focused on constructive feedback, can strengthen self-efficacy and enhance learning outcomes [74] as reported by the students in the BIP. The students also noted that personal interactions outside the university setting helped to reduce feelings of insecurity regarding their knowledge and skills, making participation in simulations less daunting. Humphreys asserts that successful learning outcomes from simulations depend on the ability of educators to promote peer group cohesion prior to the activity [77]. Informal interactions between teachers and students have also shown to significantly influence students’ learning outcomes [71] and observations and verbal encouragement from others, as well as interprofessional interaction, are key sources of self-efficacy development [59, 73]. All of which underlines the importance of both the non-academic start of the face-to-face part of the BIP and the major contributing role of the educators in the students’ learning.

### Trustworthiness and limitations of the study

The study employed a qualitative research approach, using focus group discussions to gain insights into the students’ motivation for, and experiences from, participating in the BIP. The methodology possesses certain strengths and weaknesses. The decision to use teachers who had participated in the intervention as interviewers may have offered unique advantages because of their familiarity with the programme and the participants. Insight in the programme may have made it easier to ask follow-up questions. However, the dual role of teacher-interviewers may also introduce potential bias [78]. As participants in the intervention, these teachers may have held preconceived notions or expectations regarding the programme’s impact, which could unintentionally influence how questions were framed or how responses were interpreted. The potential for bias in data collection may have had impact to trustworthiness and rigor of the findings as the interviewers’ personal experiences with the programme could have shaped their approach to both the interviews and analysing responses [26]. Notably, one interviewer had not participated in the intervention, potentially contributing to reduce this potential bias.

Focus group interviews provide valuable insights but also have notable limitations. Group dynamics can influence responses, with dominant participants potentially overshadowing others, making it difficult for more reserved individuals to share their views, especially in a group setting. Individual interviews can supplement focus groups to provide a broader range of items [79] and deeper insights into reluctant topics, such as personal feelings, opinions and experiences [80]. Despite this, the interviewers did not perceive that anything related to the topics or the research objective had been overlooked. However, these considerations should be taken into account in future research to further refine the methodology and enhance data reliability.

The theme guide was developed based on the experiences of its developers and prior knowledge from relevant research. It is recommended that, when creating a theme guide, one should acquire a comprehensive and adequate understanding of the subject so that previous research serves as a framework for the interview. Such an acquisition of knowledge on the topic can be achieved by conducting an extensive literature review [81]. This was not done in the present study. In retrospect, this might have led to different questions and a different focus in the guide, which in turn could have led to different results. The data analysis was conducted exclusively by team members from one of the participating institutions. However, to enhance trustworthiness, all authors reviewed the transcripts and analyses, ensuring a broader and more comprehensive interpretation. In hindsight, the development of a codebook would likely have improved intercoder reliability by ensuring a shared understanding of code definitions and application across coders.

The focus group interviews were conducted in English, which was not the native language of any of the participants. This may have resulted in the participants feeling less free to articulate themselves in a non-native language as they would have in their mother tongue, potentially affecting the depth and authenticity of the data [82]. This issue could have been avoided by organising focus groups with participants from only one country, allowing the interviews to be conducted in the participants’ native language. However, this approach might undermine one of the key advantages of focus group interviews, namely the diversity of participants, which gives a richness and variety in the perspectives shared.

## Conclusion

This study provides valuable insights into the motivations and experiences of students who chose to participate in a short-term BIP focused on MCI. The study highlights that students were primarily motivated by a strong interest in the field of study and a desire to engage in international encounters, aiming to broaden their personal and professional horizons. Furthermore, it underscores the potential of short-term programmes to facilitate the acquisition of cultural and academic competencies through immersive and targeted experiences, combining online preparation with face-to-face participation abroad.

Moreover, the BIP enabled students to expand their perspective on nursing in providing emergency care as a health professional and receiving care as an injured patient, prompting reflection on delivering empathetic care and effectively communicating with patients, even across language barriers. Practical training alongside professionals responsible for pre-hospital triage, life-supporting activities and emergency trauma care enriched students’ understanding and skills in interprofessional cooperation. While students faced challenges such as language barriers and professional discrepancies, they expressed that coping with these barriers was an enhancement to their learning experience and led to increased self-confidence, improved language skills, reduced uncertainty in acute situations and personal growth. These findings are in alignment with previous research as discussed, which is encouraging as it implicates that a BIP can lead to learning outcomes comparable to longer exchange periods. Shorter exchange programmes seem to be more accessible contributing to achieving some of the goals of the Erasmus + programme for student mobility.

### Implication for practice and future research

The findings of this study underscore the transformative potential of short-term exchange programmes in nursing education. Integrating BIPs into the curriculum can be an effective strategy to develop global health competencies among students. Interprofessional collaboration and SBL seem to prepare students for real-world healthcare challenges. Additionally, fostering language proficiency, academic knowledge and cultural sensitivity through preparatory sessions can help mitigate common barriers related to short-time exchange programmes, enhancing the overall learning experience. A safe learning environment between peers and teachers should be emphasised throughout the programme, including the digital sessions prior to the face-to-face component.

However, several key areas for further research and development remain. These include ensuring sustainable learning outcomes, aligning global and professional goals and addressing barriers to participation. To maximise the effectiveness of BIPs, institutions should adopt longitudinal assessments, strengthen intercultural training and customise programme content to accommodate the diverse needs of participants. Additionally, further research is vital to assess short-term exchange programmes’ long-term benefits on professional development, including study abroad programmes’ effects on future employment outcomes.

## Electronic supplementary material

Below is the link to the electronic supplementary material.

Supplementary Material 1

## Data Availability

The data generated and analysed during the current study are not publicly available due to anonymization. However, the transcriptions and the analysis table are available from the corresponding author upon reasonable request.

## Abbreviations

ABCDE: Airways, Breathing, Circulation, Disability and Exposure
BIP: Blended Intensive Programme
CPR: Cardiopulmonary Resuscitation
CV: Curriculum Vitae
ECTS: European Credit and Accumulation System
MCI: Mass Casualty Incident
SBL: Simulation-Based Learning
TBL: Team-Based Learning
