# Supplementary material for: Nursing students' motivation for, and experiences from, participating in a blended intensive programme on mass casualty incidents: a qualitative study
Source: BMC Med Educ. 2025 Jun 4;25:836. doi: 10.1186/s12909-025-07425-y (PMC12139066; doi:10.1186/s12909-025-07425-y)
Supplement: Supplementary file 1 — Supplementary Material 1 [file 12909_2025_7425_MOESM1_ESM.pdf]

Theme guide for focus group interview - Blended intensive programme focusing on mass casualty incidents

Brief introduction to the study, audio recording, transcription, and participants' voluntary participation and rights to withdraw from the study.

What was the motivations for participating

Previous experience with study abroad programme

How students generally experienced being part of the exchange programme

How it felt to meet and collaborate with nursing students from other countries

How it felt to come to Norway as a nursing student

What academic benefits they gained from participating in the exchange programme

What non-academic benefits they gained from participating in the exchange programme

What were the negative aspects of participating in the exchange programme, if any

What challenges did they encounter (language barrier, cultural diversities, academic differences)

Suggestions for further improvements of the programme
